# Optimal systolic and diastolic blood pressure threshold that associated with lower risk of white matter hyperintensity progression

**DOI:** 10.3389/fnagi.2023.1254463

**Published:** 2023-10-19

**Authors:** Sibo Liu, Mengxing Wang, De’an Gu, Yanzhao Li, Xin Zhang, Hang Li, Chenhua Ji, Ximing Nie, Jinjie Liu

**Affiliations:** ^1^Intensive Care Unit, Dalian Municipal Central Hospital Affiliated Dalian University of Technology, Dalian, China; ^2^China National Clinical Research Center for Neurological Diseases Beijing China, Beijing, China; ^3^Department of Neurology, Affiliated Qingdao Central Hospital of Qingdao University, Qingdao Cancer Hospital, Qingdao, China; ^4^Department of Neurosurgery, Affiliated Zhongshan Hospital of Dalian University, Dalian, China; ^5^Department of General Medicine, Dalian Municipal Central Hospital Affiliated Dalian University of Technology, Dalian, China; ^6^Department of Geriatrics, Affiliated Dalian Friendship Hospital of Dalian Medical University, Dalian, China; ^7^Neurocritical Care Unit, Department of Neurology, Beijing Tiantan Hospital, Capital Medical University, Beijing, China

**Keywords:** blood pressure, blood pressure variability, systolic blood pressure, diastolic blood pressure, white matter hyperintensity

## Abstract

**Background:**

The optimal control thresholds for systolic blood pressure (SBP) and diastolic blood pressure (DBP) in patients with white matter hyperintensity (WMH) are still unclear.

**Method:**

A longitudinal retrospective study of patients with brain magnetic resonance imaging (MRI) scans with intervals of more than 3  years was conducted. Blood pressure records during hospitalization and from outpatient visits between baseline and the last MRI scan were collected. The outcome was the change in total WMH from baseline to the final visit.

**Results:**

Among the 965 patients with MRI scans, 457 patients with detailed longitudinal blood pressure records were ultimately included and classified into the WMH absent group (*n* = 121), mild WMH group (*n* = 126), and moderate to severe WMH group (*n* = 210). Both baseline and longitudinal mean SBP, DBP, and SBP SD were significantly associated with WMH severity (*p* < 0.05). An average SBP of 130-140 mmHg [vs. <130 mmHg, aOR, 1.80, (95% CI, 1.05–3.07), *p* = 0.03] was associated with a higher risk of WMH progression. DBP ≥ 90 mmHg [vs. <80 mmHg, OR, 1.81, (95% CI, 0.88–3.74), *p* = 0.02, aOR, 1.54, (95% CI, 0.66–3.53), *p* = 0.32] was associated with a higher risk of WMH progression, but was not after adjusted for other covariates. Longitudinal BP variability was not significantly associated with WMH progression.

**Conclusion:**

Both SBP and DBP had a stronger relationship with the severity of WMH. A target mean SBP of <130 mmHg and mean DBP of <80 mmHg was associated with a lower risk of WMH progression.

## 1. Introduction

White matter hyperintensity (WMH) is an important risk factor for cognitive dysfunction (Hu et al., 2021) and neuropsychiatric symptoms (Clancy et al., 2021) worldwide. Two meta-analyses have indicated a strong relationship between high blood pressure (BP) and the severity of WMH, especially in elderly individuals (Alateeq et al., 2021; Wilkinson and Webb, 2022). After adjusting for age, hypertension was the strongest risk factor for the development of WMH (Liao et al., 1996; Basile et al., 2006). Patients with uncontrolled hypertension have a higher risk of severe WMH and also face a higher risk of WMH progression compared with those who accepted hypertension prevention or treatment (Dufouil et al., 2001; Verhaaren et al., 2013). Additionally, patients with hypertension tend to have early occurrence of WMH (de Leeuw et al., 2002).

Relatively fewer studies have focused on the threshold of BP control in patients with WMH. An observational study revealed that a systolic blood pressure (SBP) higher than 160 mmHg or lower than 140 mmHg was associated with higher risk of WMH progression compared to an SBP between 140 and 160 mmHg in patients over 80 years old (Peng et al., 2014), indicating that an excessively high or low SBP contributes to the development of WMH in special populations. Previously reported studies used the traditional SBP threshold of <140 mmHg, and had a mean SBP over 130 mmHg after treatment (de Leeuw et al., 2002; Dufouil et al., 2005). In 2019, the Systolic Blood Pressure Intervention Trial (SPRINT), a multicenter randomized controlled study (RCT) with a 4-year follow-up (Nasrallah et al., 2019), included 670 patients older than 50 years without a history of diabetes and stroke who were randomly classified into two groups: (1) the intensive intervention group with an SBP less than 120 mmHg and (2) the standard treatment group with an SBP less 140 mmHg. The results of this study showed that among hypertensive adults, targeting SBP of less than 120 mmHg was significantly associated with a slower development of WMH (Nasrallah et al., 2019). However, at the end of intervention, the mean SBP was 120.7 mmHg in the intensive treatment group vs. 134.9 mmHg in the standard treatment group (Nasrallah et al., 2019). In 2020, a longitudinal study enrolled 505 community-dwelling and cognitively normal elderly individuals and measured BP values three times in a sitting position. The study revealed that a low SBP, defined as an SBP ≤110 mmHg, was independently associated with the severe periventricular WMH in patient with controlled hypertension but not in those without hypertension (Kim et al., 2020). High SBP variability was also shown to have a strong relationship with WMH severity and development (Zhang et al., 2022). Therefore, what is the optimal mean SBP and SBP variability that should be targeted to stop or slow the development of WMH?

Currently, whether diastolic blood pressure (DBP) plays an important role in the development and progression of WMH remains controversial (Zhang et al., 2020; Wilkinson and Webb, 2022). Several studies have supported a stronger association between DBP and WMH, compared to SBP. In 2011, a Northern Manhattan study (Marcus et al., 2011) including 1,290 stroke-free participants analyzed the association of two recorded BP values with changes in WMH from baseline to the last MRI scan and showed that the baseline DBP value and the longitudinal increase in DBP were independently associated with a greater increase in WMH volume (Marcus et al., 2011). Further literature from the Northern Manhattan study also indicated that DBP was associated with the region-specific load of WMH. Compared to patients with a DBP higher than 90 mmHg, those with a DBP between 80 and 89 mmHg presented a WMH volume in the anterior periventricular region that was approximately 12% lower and a WMH volume in the posterior periventricular region that was 9% lower; however, SBP values were not significantly associated with the regional location of WMH (Caunca et al., 2020). The mean age of the participants in these two studies was 64 ± 8 years, ranging from 40 to 90 years (Marcus et al., 2011; Caunca et al., 2020). A previous meta-analysis showed that the association of DBP with WMH was closer than that of SBP with WMH, and the risk of WMH progression in patients under 70 years old was higher than that in patients over 70 years old when the DBP was increased (Zhang et al., 2020). Data from a prospective, community-based, multicenter cohort study (UK Biobank) of midlife patients between 40 to 69 years old also revealed that although WMH was more strongly associated with current SBP control, past DBP control had a stronger association with WMH load compared to past SBP control, especially in patients under 50 years old (Wartolowska and Webb, 2021). These studies indicated a more important role of DBP in the occurrence and development of WMH in midlife patients, in whom WMH starts to appear earlier among those with hypertension (de Leeuw et al., 2002). Although, low DBP, defined as a DBP <60 mmHg, did not show an association with WMH severity (Kim et al., 2020). As an important factor that determines organ perfusion, a low DBP was reported to be the only independent risk factor associated with periprocedural stroke or death (de Waard et al., 2017) and was associated with an increased risk of cardiovascular events (Stensrud and Strohmaier, 2017; Böhm et al., 2018; Tsujimoto and Kajio, 2018). Greater DBP variability and a low DBP have also been confirmed as risk factors for cognitive decline in later life (average 62.5 years old) (Lee et al., 2020; Peters and Xu, 2022; Zhang et al., 2022). However, the optimal control thresholds for the mean DBP and DBP variability in patients with WMH still remains unclear.

To provide more evidence for SBP and DBP control in patients with WMH, this study mainly focused on longitudinal SBP and DBP data from baseline to the final MRI scan. Second, in this study, we aimed to determine the optimal threshold of mean SBP and DBP, and BP variability associated with a lower risk of WMH progression.

## 2. Method

### 2.1. Study design and patients

This was a longitudinal retrospective study involving patients admitted to Dalian Central Municipal Hospital between January 1st, 2008, and January 1st, 2022, with dynamic MRI scans with intervals of more than 3 years and detailed blood pressure records between the baseline and last magnetic resonance imaging (MRI) both in hospitalization and outpatient visits. The inclusion criteria were as follows: (1) age over 18 years old; (2) BP records of more than 5 times every year. The exclusion criteria were as follows: (1) WMH with a defined or suspicious diagnosis of gene disease such as cerebral autosomal dominant arteriopathy with subcortical infarcts and leukoencephalopathy (CADASIL); (2) WMH with a defined or suspicious diagnosis of acute inflammation or immune-mediated small vessel disease; (3) patients with large area cerebral infarction that influenced the analysis of WMH; and (4) patients without detailed clinical data.

### 2.2. Demographic and clinical data collection

Baseline demographic variables, including age, sex, and education, were collected. Clinical variables, including history of hypertension, diabetes mellitus, hyperlipidemia, hyperhomocysteinemia, smoking, alcohol consumption, cardiovascular disease, atrial fibrillation, heart failure, kidney dysfunction, chronic respiratory disease, autoimmune disease, thyroid disease, anxiety, and depression, were all collected. The average interval of MRI from baseline to the last scans was calculated between groups. Etiology of hospitalization were also collected.

### 2.3. Blood pressure collection and value calculation

BP values were collected from inpatient records (daily monitoring of BP in the upper arm while in the sitting position during hospitalization), outpatient visit records, community monitoring records, and home monitoring records (from community personal files and past medical records). BP values were calculated as baseline mean SBP and DBP values (mmHg), mean SBP and DBP values for longitudinal control (mmHg), and two commonly used BP variability indices, including SDs and coefficients of variation (CVs). The intervals of mean SBP were stratified into <130 mmHg, 130–140 mmHg, 140–150 mmHg, and ≥ 150 mmHg; the intervals of mean DBP were stratified into <80 mmHg, 80–90 mmHg, and ≥ 90 mmHg. BP variability were calculated using the SD (mmHg), and CV (CV=SD/mean×100, %) (Zhang et al., 2022). BP SD and CV were divided into low, median, and high variability groups using fuzzy C-means (FCM) algorithm (Yang et al., 2022).

### 2.4. White matter hyperintensity assessment

All patients had undergone dynamic MRI scans (Philips Achieva 3.0 T magnetic resonance system, Philips Healthcare, United States, 5 mm thick slices). The presence and severity of WMH was assessed based on the baseline and last MRI by two neuroimaging specialists according to the semiquantitative Fazekas scale (Iordanishvili et al., 2019; Nasrallah et al., 2019). If the assessment of both were the same, the results were obtained. If the results for both were the same, the data were entered. If the results were not the same, the third independent neuroimaging specialist choose and entered the “more accurate” result. Periventricular and deep WMHs were rated separately according to the Fazekas scale. Periventricular WMH was graded as follows: grade 0 for no lesions; grade 1 for caps or pencil-thin lining; grade 2 for smooth halo; and grade 3 for irregular periventricular WMH extending into a deep WMH. Deep WMH was graded as follows: grade 0 for no lesions; grade 1 for punctate foci; grade 2 for beginning confluence of foci; and grade 3 for large fused areas. A total Fazekas score, ranging from 0 to 6, was acquired by summing the periventricular and deep WMH scores. The severity of WMH was categorized as absent (total Fazekas score 0), mild (total Fazekas score 1–2), and moderate to severe (total Fazekas score 3–6). The progression of WMH was calculated as an increase in the total Fazekas score every 3 years from baseline to the last MRI [Progression = (total Fazekas score at last visit minus total Fazekas score at baseline)/(time interval/3 years)]. Stable WMH was defined as a total Fazekas increase = 0 every 3 years; progression was defined as a total Fazekas increase≥0 every 3 years; slow progression was defined as a total Fazekas increase = 0–1 every 3 years; and rapid progression was defined as a total Fazekas increase ≥1 every 3 years. Stable and progression of WMH was used to analyze the association between BP values and the risk of WMH progression. Stable, slow progression and rapid progression of WMH was used to describe the distribution of WMH progression under different threshold of BP values.

### 2.5. Statistical analysis

Baseline characteristics of the study population, categorized based on the severity of WMH, were compared with standard statistics. Continuous variables are expressed as the mean (SD) or median (interquartile range, IQR). Categorical variables are presented as counts (percentages). Fisher’s exact tests or chi-square tests were used for categorical variables. ANOVA or Kruskal–Wallis tests were used for continuous variables. Ordinal logistic analysis was used to evaluate the associations between different BP values and the severity of WMH. Binary logistic regression was used to analyze the association between different BP thresholds and the risk of WMH progression, of which Model 1 was not adjusted and Model 2 was adjusted for age, education level, diabetes mellitus, hyperhomocysteinemia, smoking status, stroke, kidney dysfunction, and etiology of hospitalization.

A two-sided *p* value <0.05 was considered significant. All statistical analyses were performed using SAS version 9.4 (SAS Institute Inc., Cary, NC).

## 3. Results

### 3.1. Demographic and clinical characteristics

**Figure 1:**
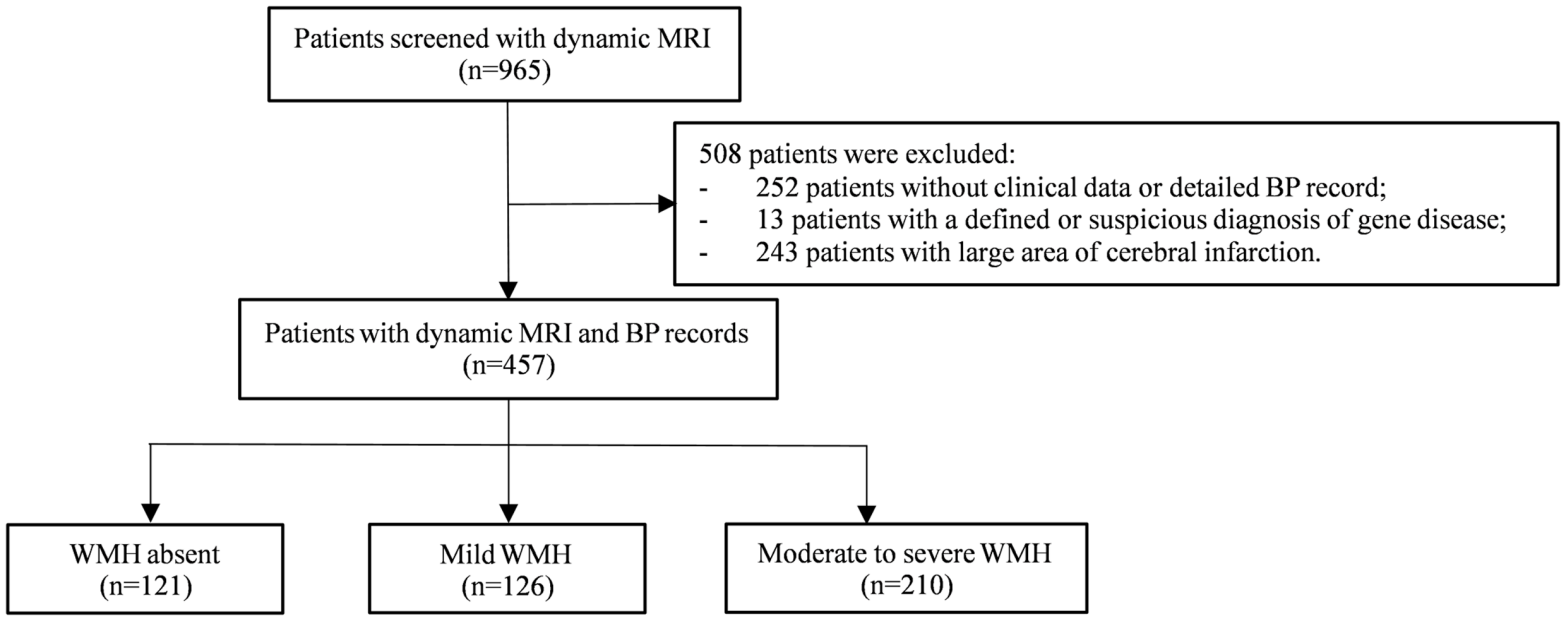
Flow chart. MRI, magnetic resonance imaging; BP, blood pressure; WMH, white matter hyperintensity.

A total of 965 patients with MRI scans with an interval of more than 3 years were screened, among whom 457 were ultimately included in the following analysis and were further categorized into the WMH absent group (*n* = 121), mild WMH group (*n* = 126), and moderate to severe WMH group (*n* = 210) (Figure 1). Demographic and clinical characteristics are shown in Table 1. The mean age at baseline was 61.79 ± 8.11 years in total, 57.93 ± 8.54 in the WMH absent group, 62.72 ± 6.14 years in the mild WMH group, and 63.45 ± 8.19 years in the moderate to severe WMH group (*p* < 0.001). Other risk factors with significant differences between groups were education level, hypertension, diabetes mellitus, hyperhomocysteinemia, smoking status, history of stroke, kidney dysfunction. The mean intervals of MRI from baseline to the last scan were 6.52 ± 2.97 years in total, 6.83 ± 2.90 years in the WMH absent group, 6.47 ± 2.83 years in the mild WMH group, and 6.38 ± 3.09 years in the moderate to severe WMH group (*p* = 0.24). Etiology of hospitalization were also different between groups, especially hospitalization due to cerebral vascular disease (*p* < 0.001). The median number of BP measurement time points was 16 (de Leeuw et al., 2002; Dufouil et al., 2005; Marcus et al., 2011; Peng et al., 2014; de Waard et al., 2017; Stensrud and Strohmaier, 2017; Böhm et al., 2018; Tsujimoto and Kajio, 2018; Nasrallah et al., 2019; Caunca et al., 2020; Kim et al., 2020; Lee et al., 2020; Zhang et al., 2020, 2022; Wartolowska and Webb, 2021; Peters and Xu, 2022).

**Table 1:** Baseline characteristics.

	**Total (*n* = 457)**	**WMH absent (*n* = 121)**	**Mild WMH (*n* = 126)**	**Moderate to severe WMH (*n* = 210)**	***p* value**
**Demographic variables**					
Age at baseline, mean (SD)	61.79 ± 8.11	57.93 ± 8.54	62.72 ± 6.14	63.45 ± 8.19	<0.001
Sex (Male, *n*%)	234 (51.2)	61 (50.41)	63 (50.00)	110 (52.38)	0.90
Higher education (*n*, %)	86 (18.82)	33 (27.27)	26 (20.63)	27 (12.86)	0.005
**Clinical variables**					
Hypertension (*n*, %)	130 (28.45)	48 (39.67)	48 (38.10)	34 (16.19)	<0.001
Diabetes mellitus (*n*, %)	292 (63.89)	89 (73.55)	82 (65.08)	121 (57.62)	0.01
Hyperlipidemia (*n*, %)	232 (50.77)	69 (57.02)	65 (51.59)	98 (46.67)	0.19
HCY (*n*, %)	127 (27.79)	19 (15.70)	26 (20.63)	82 (39.05)	<0.001
Smoke (*n*, %)	141 (30.85)	25 (20.66)	36 (28.57)	80 (38.10)	0.003
Alcohol (*n*, %)	86 (18.82)	18 (14.88)	23 (18.25)	45 (21.43)	0.33
Stroke (*n*, %)	104 (22.76)	3 (2.48)	12 (9.52)	89 (42.38)	<0.001
CAD (*n*, %)	87 (19.04)	21 (17.36)	25 (19.84)	41 (19.52)	0.86
Atrial fibrillation (*n*, %)	24 (5.25)	8 (6.61)	8 (6.35)	8 (3.81)	0.44
Heart failure (*n*, %)	5 (1.09)	3 (2.48)	0 (0.00)	2 (0.95)	0.17
Kidney dysfunction (*n*, %)	19 (4.16)	1 (0.83)	4 (3.17)	14 (6.67)	0.03
Chronic respiratory disease (*n*, %)	19 (4.16)	6 (4.96)	6 (4.76)	7 (3.33)	0.72
Autoimmune disease (*n*, %)	1 (0.22)	0 (0.00)	0 (0.00)	1 (0.48)	0.56
Thyroid disease (*n*, %)	19 (4.16)	6 (4.96)	6 (4.76)	7 (3.33)	0.72
Anxiety (*n*, %)	31 (6.78)	8 (6.61)	5 (3.97)	18 (8.57)	0.27
Depression (*n*, %)	16 (3.50)	3 (2.48)	3 (2.38)	10 (4.76)	0.40
Interval of MRI from baseline to final, *y*, mean (SD)	6.52 ± 2.97	6.83 ± 2.90	6.47 ± 2.83	6.38 ± 3.09	0.24
Etiology of hospitalization, (*n*, %)					<0.001
Cognitive dysfunction	26 (5.69)	0 (0.00)	5 (3.97)	21 (10.00)	
Cerebral vascular disease	173 (37.85)	19 (15.70)	39 (30.95)	115 (54.76)	
Neuro-degenerative disease	46 (10.07)	21 (17.36)	14 (11.11)	11 (5.24)	
Peripheral vertigo	20 (4.38)	4 (3.31)	9 (7.14)	7 (3.33)	
Others	180 (39.39)	73 (60.33)	57 (45.24)	50 (23.81)	
Number of BP measurements, median (IQR)	16 (9–24)	15 (10–24)	15 (9–24)	16 (10–25)	1.00

WMH, white matter hyperintensity; HCY, hyperhomocysteinemia; CAD, coronary artery disease; MRI, magnetic resonance imaging.

### 3.2. Association between baseline SBP, DBP, and severity of WMH

The mean baseline SBP was 129.25 ± 10.52 mmHg in the WMH absent group, 131.33 ± 11.32 mmHg in the mild WMH group, and 141.03 ± 13.72 mmHg in the moderate to severe WMH group [aOR, 1.05, (95%CI, 1.03–1.07), *p* < 0.001, Table 2]. The mean baseline DBP was 78.85 ± 5.73 mmHg in the WMH absent group, 78.23 ± 7.50 mmHg in the mild WMH group, and 84.36 ± 7.99 mmHg in the moderate to severe WMH group [aOR, 1.07, (95%CI, 1.04–1.10), *p* < 0.001, Table 2]. Baseline BP variability including SBP SD, SBP CV and DBP SD was gradually increased with the severity of WMH, but only SBP SD and SBP CV were significant after adjustment for other covariates (Table 2).

**Table 2:** Association between SBP, DBP, and severity of WMH.

BP variables	WMH absent (*n* = 121)	Mild WMH (*n* = 126)	Moderate-to-severe WMH (*n* = 210)	Unadjusted		Adjusted	
				OR (95%CI)	*p* value	aOR (95%CI)	*p* value
Baseline BP variables							
Mean SBP, mmHg, mean (SD)	129.25 ± 10.52	131.33 ± 11.32	141.03 ± 13.72	1.07 (1.05–1.08)	<0.001	1.05 (1.03–1.07)	<0.001
SBP SD, mmHg, mean (SD)	7.68 ± 5.30	8.58 ± 5.94	11.06 ± 7.73	1.07 (1.04–1.10)	<0.001	1.04 (1.01–1.08)	0.009
SBP CV, %, mean (SD)	5.80 ± 3.97	6.43 ± 4.27	7.75 ± 5.37	1.08 (1.04–1.12)	<0.001	1.05 (1.01–1.09)	0.03
Mean DBP, mmHg, mean (SD)	78.85 ± 5.73	78.23 ± 7.50	84.36 ± 7.99	1.09 (1.06–1.12)	<0.001	1.07 (1.04–1.10)	<0.001
DBP SD, mmHg, mean (SD)	5.22 ± 3.90	6.02 ± 3.66	6.48 ± 4.76	1.06 (1.01–1.10)	<0.001	1.03 (0.98–1.08)	0.28
DBP CV, %, mean (SD)	6.68 ± 5.17	7.71 ± 4.58	7.67 ± 5.58	1.03 (0.99–1.06)	0.13	1.01 (0.97–1.05)	0.70
Longitudinal BP variables							
Mean SBP, mmHg, mean (SD)	129.04 ± 8.34	131.65 ± 9.96	140.68 ± 11.27	1.09 (1.07–1.11)	<0.001	1.08 (1.05–1.10)	<0.001
SBP SD, mmHg, mean (SD)	10.40 ± 3.85	11.28 ± 4.49	14.77 ± 14.31	1.13 (1.08–1.17)	<0.001	1.07 (1.02–1.11)	0.005
SBP CV, %, mean (SD)	8.03 ± 2.90	8.48 ± 3.16	10.25 ± 8.03	1.13 (1.08–1.20)	<0.001	1.06 (1.00–1.12)	0.07
Mean DBP, mmHg, mean (SD)	78.08 ± 4.64	77.91 ± 5.11	83.38 ± 6.65	1.15 (1.11–1.18)	<0.001	1.15 (1.10–1.20)	<0.001
DBP SD, mmHg, mean (SD)	7.14 ± 2.50	7.23 ± 2.62	8.69 ± 3.24	1.18 (1.11–1.26)	<0.001	1.11 (1.04–1.19)	0.003
DBP CV, %, mean (SD)	9.15 ± 3.27	9.26 ± 3.18	10.38 ± 3.74	1.10 (1.04–1.15)	<0.001	1.05 (1.00–1.11)	0.08

SBP, systolic blood pressure; DBP, diastolic blood pressure; WMH, white matter hyperintensity. Adjusting factors: age, education, diabetes mellitus, hyperhomocysteinemia, smoke, stroke, kidney dysfunction, and etiology of hospitalization.

### 3.3. Association between longitudinal SBP, DBP, and severity of WMH

Longitudinal mean SBP was 129.04 ± 8.34 mmHg in the WMH absent group, 131.65 ± 9.96 mmHg in the mild WMH group, and 140.68 ± 11.27 mmHg in the moderate to severe WMH group [aOR, 1.08, (95%CI, 1.05–1.10), *p* < 0.001, Table 2]. The mean longitudinal DBP was 78.08 ± 4.64 mmHg in the WMH absent group, 77.91 ± 5.11 mmHg in the mild WMH group, and 83.38 ± 6.65 mmHg in the moderate to severe WMH group [aOR, 1.15, (95%CI, 1.10–1.20), *p* < 0.001, Table 2]. The BP variability of SBP SD and DBP SD were significantly associated with the severity of WMH after adjusted (*p* < 0.05, Table 2). SBP CV and DBP CV were higher in patients with severe WMH comparing to that in patients without and with mild WMH (*p* < 0.001), but were not significant after adjustment for other covariates (*p* > 0.05, Table 2).

### 3.4. Optimal SBP and DBP thresholds associated with a lower risk of WMH progression

**Figure 2:**
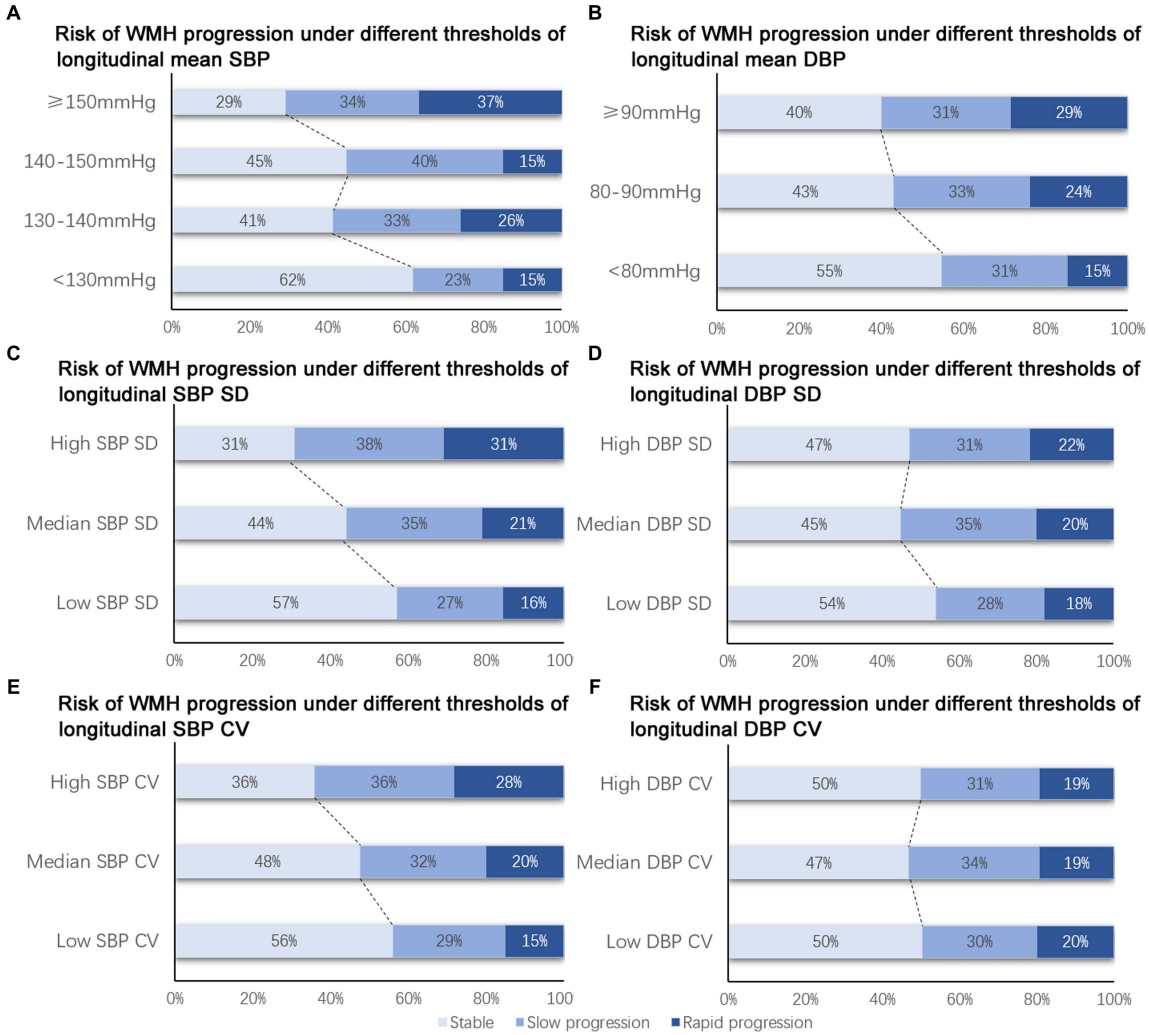
Risk of WMH progression under different BP thresholds. **(A, C, E)** Risk of WMH progression was higher with the increase of longitudinal mean SBP **(A)**, SBP SD **(B)** and SBP CV **(C)**. **(B, D, F)** Non-significant trend was observed in the risk of WMH progression with the increase of longitudinal mean DBP **(B)**, DBP SD **(D)**, DBP CV **(F)**. SBP, systolic blood pressure; DBP, diastolic blood pressure; WMH, white matter hyperintensity.

For longitudinal BP values, only mean SBP and mean DBP was associated with the risk of WMH progression, while SBP SD, SBP CV, DBP SD, and DBP CV did not showed significant relationship with the progression of WMH both before and after adjusted for other covariates (Supplementary Table S1). Compared with patients with a mean SBP lower than 130 mmHg, patients with a mean SBP of 130–140 mmHg [aOR, 1.80, (95% CI, 1.05–3.07), *p* = 0.03, Table 3], a mean SBP of 140–150 mmHg (aOR, 1.84, [95% CI, 1.00–3.38], *p* = 0.049, Table 3), and an SBP over 150 mmHg (aOR, 2.84, [95% CI, 1.20–6.70], *p* = 0.02, Table 3) showed a significantly higher risk of WMH progression (Figure 2A). However, patients with a DBP over 90 mmHg had a higher risk of WMH progression (Figure 2B) in unadjusted analysis (OR, 1.81, [95% CI, 0.88–3.74], *p* = 0.02, Table 3) compared with those with a DBP lower than 80 mmHg, but the difference remained nonsignificant after adjusting for age, education level, diabetes mellitus, hyperhomocysteinemia, smoking status, stroke, kidney dysfunction, and etiology of hospitalization [aOR, 1.54, (95% CI, 0.66–3.53), *p* = 0.32, Table 3]. A median SBP SD >11.1 mmHg [OR, 1.68, (95% CI, 1.34–2.47), *p* = 0.009, Table 3; Figure 2C] and a high SBP CV >12.24% [OR, 2.30, (95% CI, 1.26–4.11), *p* = 0.006, Table 3; Figure 2E] was significantly associated with WMH progression, but was not after adjusted. DBP variability was not associated with WMH progression both before and after adjusted (Table 3; Figures 2D,F).

**Table 3:** Optimal longitudinal SBP and DBP threshold that associated with low risk of WMH progression.

BP variables	Stable vs. Progression			
	Model 1		Model 2	
	OR	*p* value	aOR	*p* value
Mean SBP, mmHg				
<130, *n* (%)	Ref.	–	Ref.	–
130–140, *n* (%)	2.00 (1.27–3.14)	0.003	1.80 (1.05–3.07)	0.03
140–150, *n* (%)	2.30 (1.40–3.79)	0.001	1.84 (1.00–3.38)	0.049
≥150, *n* (%)	3.91 (1.86–8.22)	<0.001	2.84 (1.20–6.70)	0.02
SBP SD				
Low SBP SD	Ref.	–	Ref.	–
Median SBP SD	1.68 (1.34–2.47)	0.009	1.32 (0.87–2.03)	0.20
High SBP SD	2.97 (1.46–6.03)	0.003	1.90 (0.88–4.08)	0.10
SBP CV				
Low SBP CV	Ref.	–	Ref.	–
Median SBP CV	1.40 (0.93–2.10)	0.11	1.16 (0.75–1.79)	0.51
High SBP CV	2.30 (1.26–4.11)	0.006	1.61 (0.85–3.03)	0.14
Mean DBP, mmHg				
<80, *n* (%)	Ref.	–	Ref.	–
80–90, *n* (%)	1.60 (1.08–2.35)	0.11	1.51 (0.96–2.36)	0.07
≥90, *n* (%)	1.81 (0.88–3.74)	0.02	1.54 (0.66–3.53)	0.32
DBP SD				
Low DBP SD	Ref.	–	Ref.	–
Median DBP SD	1.45 (0.96–2.18)	0.08	1.12 (0.72–1.75)	0.62
High DBP SD	1.31 (0.79–2.20)	0.30	0.95 (0.54–1.67)	0.84
DBP CV				
Low DBP CV	Ref.	–	Ref.	–
Median DBP CV	1.45 (0.75–1.75)	0.52	0.94 (0.60–1.48)	0.79
High DBP CV	1.01 (0.62–1.66)	0.96	0.74 (0.44–1.26)	0.27

SBP, systolic blood pressure; DBP, diastolic blood pressure; WMH, white matter hyperintensity; BP, blood pressure. Model 1: unadjusted; Model 2: adjusted for age, education, diabetes mellitus, hyperhomocysteinemia, smoke, stroke, kidney dysfunction, and etiology of hospitalization. Low SBP SD: ≤11.15 mmHg; median SBP SD: 11.15–18.87 mmHg; high SBP SD:>18.87 mmHg; low SBP CV: ≤7.75%; median SBP CV: 7.75–12.24%; high SBP CV: >12.24%; Low DBP SD: ≤ 6.80 mmHg; median DBP SD: 6.80–10.18 mmHg; high DBP SD: >10.18 mmHg; Low DBP CV: ≤8.18%; median DBP CV: 8.18–12.00%; high DBP CV: >12.00%.

### 3.5. Subgroup analysis of optimal SBP and DBP thresholds associated with a lower risk of WMH progression

**Figure 3:**
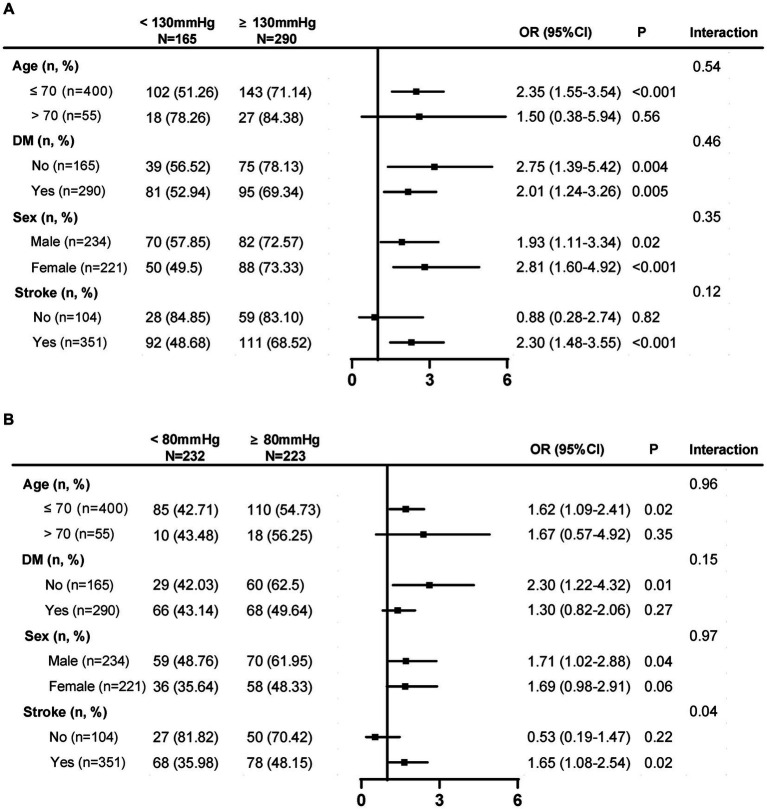
Subgroup analysis of SBP and DBP threshold that associated with a lower risk of WMH progression. **(A)** Longitudinal mean SBP lower than 130 mmHg was significantly associated with lower risk of WMH progression in patients with ≤70 years and a history of stroke. **(B)** A longitudinal mean DBP lower than 80 mmHg was significantly associated with a lower risk of WMH progression in patients ≤ 70 years, patients without diabetes mellitus, male patients, and patients with a history of stroke. SBP, systolic blood pressure; DBP, diastolic blood pressure; WMH, white matter hyperintensity; DM, diabetes mellitus. Low SBP SD: ≤11.15 mmHg; median SBP SD: 11.15–18.87 mmHg; high SBP SD:>18.87 mmHg; low SBP CV: ≤7.75%; median SBP CV: 7.75–12.24%; high SBP CV: >12.24%; Low DBP SD: ≤ 6.80 mmHg; median DBP SD: 6.80–10.18 mmHg; high DBP SD: >10.18 mmHg; Low DBP CV: ≤8.18%; median DBP CV: 8.18–12.00%; high DBP CV: >12.00%.

Longitudinal mean SBP lower than 130 mmHg was significantly associated with lower risk of WMH progression in patients with age younger than 70 years [OR, 2.35, (95% CI, 1.55–3.54), *p* < 0.001], and patients with a history of stroke [OR, 2.30, (95% CI, 1.48–3.55), *p* < 0.001], but not in patients with age over than 70 years or without a history of stroke (Figure 3A). A longitudinal mean DBP lower than 80 mmHg was significantly associated with a lower risk of WMH progression in patients younger than 70 years [OR, 1.62, (95% CI, 1.09–2.41), *p* = 0.02], patients without diabetes mellitus [OR, 2.30, (95% CI, 1.22–4.32), *p* = 0.01], male patients [OR, 1.71, (95% CI, 1.02–2.88); *p* = 0.04], and patients with a history of stroke [OR, 1.65, (95% CI, 1.08–2.54), *p* = 0.02] but not in patients aged over 70  years, diabetic patients, female patients, or patients without a history of stroke (Figure 3B).

## 4. Discussion

In this retrospective study, both SBP and DBP values were strongly associated with the severity of WMH. A mean SBP value lower than 130 mmHg, a mean DBP value lower than 80 mmHg were associated with a lower risk of WMH progression, but only mean SBP was significant after adjusted for age, education level, diabetes mellitus, hyperhomocysteinemia, smoking status, stroke, kidney dysfunction, and etiology of hospitalization. Although longitudinal SBP variability and DBP variability were associated with the severity of WMH, but were not related to risk of WMH progression.

In the SPRINT study (Nasrallah et al., 2019), intensive SBP control of lower than 120 mmHg was significantly associated with a smaller increase in mean WMH volume compared with standard SBP control of lower than 140 mmHg, although no change in cognitive decline was observed (Liao et al., 1996). At the end of the intervention, the mean SBP value was 120.7 mmHg in the intensive treatment group vs. 134.9 mmHg in the standard treatment group. In our study, the mean longitudinal SBP value was 129.04 ± 8.34 mmHg in the WMH absence group, 131.65 ± 9.96 mmHg in the mild WMH group, and 140.68 ± 11.27 mmHg in the moderate to severe WMH group (Nasrallah et al., 2019). Patients with a mean SBP value lower than 130 mmHg showed a significantly lower risk of WMH progression compared to those with SBP values of 130–140, 140–150, and ≥ 150 mmHg. This evidence confirmed a positive effect of the intensive reduction of the mean SBP to a value lower than 130 mmHg to reduce the risk of WMH progression. As an SBP lower than 110 mmHg has also been reported to be associated with more severe periventricular WMH in patients with a history of hypertension (de Waard et al., 2017), the control of mean SBP between 110 mmHg and 130 mmHg seems to be the optimal target threshold, although further study is still needed.

Until now, the association between DBP and WMH has remained controversial. Two studies using the data from the Northern Manhattan study revealed a significant association between DBP and WMH progression (Marcus et al., 2011) and the region-specific load of WMH in the anterior periventricular region and the posterior periventricular region (Caunca et al., 2020), while a nonsignificant association was observed between SBP and both WMH progression and region distribution (Marcus et al., 2011; Caunca et al., 2020). To better reflect patients’ BP control status between baseline and the last MRI scans, we collected in our study more detailed BP records every year, including inpatient records, outpatient visit records, community monitoring records, and home monitoring records. The results of the study also showed a significant association between DBP and the severity of WMH, which was consistent with the Northern Manhattan study. A trend of elevated risk of WMH progression with increasing DBP levels was also observed in this study. The mean longitudinal DBP value was 78.08 ± 4.64 mmHg in the WMH absence group, 77.91 ± 5.11 mmHg in the mild WMH group, and 83.38 ± 6.65 mmHg in the moderate to severe WMH group. A mean DBP value lower than 80 mmHg was evidently associated with a higher risk of WMH progression in unadjusted analysis, but the association was nonsignificant after adjusting for age, education, diabetes mellitus, hyperhomocysteinemia, smoking, stroke, kidney dysfunction, and etiology of hospitalization. The DBP variability was not correlated with WMH progression before or after adjustment. A DBP value lower than 60 mmHg has been reported to increase the risk of faster cognitive decline (Razay et al., 2009), periprocedural stroke and death (de Waard et al., 2017), and cardiovascular events (Stensrud and Strohmaier, 2017; Böhm et al., 2018; Tsujimoto and Kajio, 2018) but not the severity of WMH (Kim et al., 2020). A DBP value between 60 and 80 mmHg seems to be the optimal threshold in patients with hypertension.

In our study, SBP SD, SBP CV, DBP SD, and DBP CV was not associated with the risk of WMH progression. Although SBP SD >11.1 mmHg and an SBP CV of >12.24% was associated with the risk WMH progression, but this was not significant after adjusting for age, education level, diabetes mellitus, hyperhomocysteinemia, smoking status, stroke, kidney dysfunction, and the etiology for hospitalization. Previous studies had reported an independent association between 24 h BP variability and WMH volumes/severity (van Middelaar et al., 2019; Zhang et al., 2022). There were several explanations that may be associated with the different result of our study and previous studies. Firstly, the mean age of the patients were about 70–80 years old or elder (Liu et al., 2016; van Middelaar et al., 2019; Zhang et al., 2022). However, the mean age of patients in our study was 61.79 ± 8.11 in total. Older population may be more sensitive to BP variability. Besides, in previous studies, the evaluation of MWH mainly focused on the severity or volume of the last visit (van Middelaar et al., 2019; Zhang et al., 2022), which was different from a dynamic progression of WMH in our study. Thirdly, the frequency and time interval of BP recording in our study was not that regular comparing to other studies. Further studies are still needed.

Subgroup analysis in our study revealed that the risk of WMH progression was significantly reduced when the SBP value was lower than 130 mmHg and the DBP value was lower than 80 mmHg in patients younger than 70 years old and patients with a history of stroke. This was consistent with a previous meta-analysis that indicated a higher risk of WMH progression in patients under 70 years old than in patients over 70 years old when their DBP increased (Verhaaren et al., 2013; Zhang et al., 2020). A clinical registry study (PORTYWHITE) involving neurology outpatients aged 18–55 years with vascular WMH on MRI and a score of II or III on the Fazekas scale revealed that although patients tended to be concentrated in the oldest age groups (age ≥ 45 years), patients without a stroke history were younger and had a lower burden of WMH and fewer vascular risk factors; however, hypertension was still the most frequent vascular risk factor (Viana-Baptista et al., 2019). In patients between 40 and 69 years old, past DBP control showed the strongest association with WMH risk when comparing the current DBP level and past SBP control, especially in patients under 50 years old (Wartolowska and Webb, 2021). These findings may also demonstrate potential greater benefits of strict BP control in younger patients and patients with a history of stroke, especially DBP control. In elderly patients or patients with multiple diseases, excessive lowering of SBP and DBP should be carefully considered. Our study excluded patients with large-area stroke but included patients with minor stroke and lacunes. Data from the framework of the international multisite MRI-Genetics Interface Exploration (MRI-GENIE) study showed that a higher WMH burden was associated with increased stroke severity and depended on stroke lesion locations (Bonkhoff et al., 2022). The presence of lacunes at the initial visit was a strong predictor of WMH progression (Jiang et al., 2022). For stroke type, patients with small artery occlusion had significantly higher WMH volumes compared to those with other stroke subtypes (Giese et al., 2020). The relationship between WMH and large artery sclerosis could not exclude multitype target organ damage caused by similar vascular risk factors, as earlier studies failed to determine the association between intracranial large-artery stenosis and WMH (Park et al., 2015).

Patients with hypertension tend to develop lower myelin content, have more severe brain microstructure impairment (Laporte et al., 2023a), have a higher risk of perivascular inflammation (Solé-Guardia et al., 2023), have lower cerebrovascular density, and have lower branch numbers (Zhang et al., 2021), which are potential mechanisms of WMH (Ungvari et al., 2021). Data from the Baltimore Longitudinal Study of Aging (BLSA) and the Genetic and Epigenetic Signatures of Translational Aging Laboratory Testing (GESTALT) studies analyzed the association between hypertension and myelin content measured by Diffusion tensor imaging (DTI) and showed that patients with hypertension presented lower myelin content and higher impairment to the brain microstructure, especially in the corpus callosum, fronto-occipital fasciculus, temporal lobes, internal capsules, and corona radiata (Laporte et al., 2023a). Additionally, elevated arterial stiffness was associated with lower microstructural integrity of white matter, and this association was also stronger and significant in the splenium of the corpus callosum and internal capsules, which have been reported to be sensitive to elevated arterial stiffness (Laporte et al., 2023b). Controlling arterial stiffness may represent a therapeutic target in maintaining the health of white matter tissue in the cerebral normative aging (Laporte et al., 2023b). Patients with a history of hypertension also have two times the risk of perivascular inflammation both in white matter with hyperintensity and normal-appearing white matter, indicating that neurovascular inflammation is involved in the etiology of WMH (Solé-Guardia et al., 2023). This evidence indicated that patients with hypertension are potentially susceptible to the development of WMH and that early prevention and intervention of hypertension is helpful to stop or delay WMH development.

This study had several differences and limitations compared with other studies. The first was that we used the Fazekas scale to semi-quantitatively analyze severity and WMH progression rather than WMH volume. As the Fazekas scale is a commonly used tool both in the clinic and in clinical studies, it may be easier and applicable to help clinicians understand the severity of WMH and the velocity of WMH progression. The second was that we did not analyze a lower SBP of <120 mmHg or < 110 mmHg and DBP <60 mmHg as there were very few patients with a mean SBP of lower than 120 mmHg and DBP lower than 70 mmHg in our study. The third limitation of the retrospective study was that the total number of blood pressure records and the time interval between blood pressure measurements, which might differ among different patients as the number of hospitalizations and outpatient visits were different. These blood pressure records may not be adequate to represent the patients’ average daily control of blood pressure.

## 5. Conclusion

In this longitudinal retrospective study, both SBP and DBP had a strong relationship with the severity of WMH. A target mean SBP lower than 130 mmHg and a mean DBP lower than 80 mmHg were associated with a lower risk of WMH progression. An SBP SD value <11.1 mmHg and an SBP CV value <12.24% were associated with lower risk of WMH progression, but DBP variability was not.

## Data availability statement

The original contributions presented in the study are included in the article/Supplementary material, further inquiries can be directed to the corresponding author.

## Ethics statement

All procedures were performed in accordance with relevant named guidelines and regulations and were approved by the ethics committee of the Dalian Central Municipal Hospital. Written informed consent was not required to participate in this study in accordance with the local legislation and institutional requirements.

## Author contributions

SL: Conceptualization, Data curation, Formal analysis, Funding acquisition, Investigation, Methodology, Project administration, Resources, Software, Writing – original draft. MW: Data curation, Formal analysis, Supervision, Validation, Visualization, Writing – original draft. DG: Data curation, Investigation, Validation, Writing – original draft. YL: Data curation, Investigation, Validation, Writing – original draft. XZ: Data curation, Investigation, Validation, Writing – original draft. HL: Data curation, Investigation, Validation, Writing – original draft. CJ: Investigation, Validation, Writing – original draft. XN: Investigation, Conceptualization, Data curation, Formal analysis, Funding acquisition, Methodology, Project administration, Resources, Writing – review & editing. JL: Conceptualization, Data curation, Formal analysis, Funding acquisition, Investigation, Methodology, Project administration, Writing – review & editing.
