# Supplementary material for: Optimal systolic and diastolic blood pressure threshold that associated with lower risk of white matter hyperintensity progression
Source: Front Aging Neurosci. 2023 Oct 19;15:1254463. doi: 10.3389/fnagi.2023.1254463 (PMC10620971; doi:10.3389/fnagi.2023.1254463)
Supplement: Supplementary file 1 [file Table_1.DOCX]

**Supplemented Table 1. Association between longitudinal BP values and the progression of WMH.**

| **BP variables** | Stable vs. Progression | | | |
| --- | --- | --- | --- | --- |
|  | Model 1 | | Model 2 | |
|  | OR | P value | aOR | P value |
| Mean SBP, mmHg | 1.04 (1.02-1.06) | <0.001 | 1.03 (1.01-1.05) | 0.01 |
| SBP SD | 1.01 (0.99-1.03) | 0.54 | 1.00 (0.98-1.02) | 0.64 |
| SBP CV | 1.01 (0.98-1.04) | 0.60 | 0.99 (0.96-1.03) | 0.62 |
| Mean DBP, mmHg | 1.04 (1.01-1.08) | 0.006 | 1.04 (1.00-1.08) | 0.04 |
| DBP SD | 1.01 (0.95-1.07) | 0.79 | 0.97 (0.90-1.03) | 0.32 |
| DBP CV | 0.99 (0.94-1.05) | 0.76 | 0.96 (0.91-1.02) | 0.17 |

Table 1. Association between longitudinal BP values and the progression of WMH. SBP, systolic blood pressure; DBP, diastolic blood pressure; WMH, white matter hyperintensity; BP, blood pressure. Model 1: unadjusted; Model 2: adjusted for age, education, diabetes mellitus, hyperhomocysteinemia, smoke, stroke, kidney dysfunction, and etiology of hospitalization.
